# A new species of *Perinereis* (Polychaeta, Nereididae) from Florida, USA, with a key to all *Perinereis* from the American continent

**DOI:** 10.3897/zookeys.312.4535

**Published:** 2013-06-24

**Authors:** Jesús Angel de León-González, Carrie A. Goethel

**Affiliations:** 1 Universidad Autónoma de Nuevo León, Facultad de Ciencias Biológicas, Ap. Postal 5, Suc. “F”, San Nicolás de los Garza, Nuevo León, 66451, México; 2 Ecological Associates, Inc. P. O. Box 405. Jensen Beach, Florida 34958, USA

**Keywords:** Annelida, polychaetes, Nereididae, new species, taxonomy, Florida, Western Atlantic

## Abstract

Specimens belonging to a new species of *Perinereis* Kinberg, 1865 were collected fromnatural oyster reefs in an estuarine environmenton Florida’s southwest coast. The genus *Perinereis* includes more than 70 species, of which, *Perinereis aibuhitensis* (Grube, 1878), *Perinereis brevicirrata* (Treadwell, 1920), *Perinereis camiguinoides* (Augener, 1922), *Perinereis jascooki* Gibbs, 1972, *Perinereis kuwaitensis* Mohammad, 1970, *Perinereis singaporiensis* (Grube, 1878), *Perinereis vancaurica* (Ehlers, 1868) and the new species have two short bars on Area VI and notopodial dorsal ligules that are not greatly expanded. The most geographically close species is *Perinereis brevicirrata*. The new species can be distinguished from *Perinereis brevicirrata* by the absence of a notopodial prechaetal lobe, Area V with 3 cones in a triangle, and Area VII-VIII with two well-defined rows of 33 paragnaths, the basal row having longer paragnaths in relation to the distal ones. The new species resembles *Perinereis singaporiensis* based on the absence of notopodial prechaetal lobe; however, the two species differ in some morphological characteristics such as tentacular cirri length, shape of dorsal notopodial ligules, and falciger blades. A key to all American species of *Perinereis* is included.

## Introduction

Nereididae Blainville, 1818 is probably the most well known family of Polychaeta, with 44 genera and 677 species (Read and Fauchald 2012), although the number of species could be over estimated. The importance of this family is manifested by its high diversity and abundance in practically all marine benthic environments, from the supralittoral to the abyssal depth. One of the most diverse genera in the family is *Perinereis*, established by Kinberg (1865). *Perinereis* is characterized principally by the ornamentation of pharyngeal paragnaths in Area VI, which may be simple (short or long ribbon-shaped) or fragmented transverse bars. It is a genus consisting globally of around 74 species (Read and Fauchald 2012). Bakken and Wilson (2005) found that *Perinereis* may be polyphyletic. Twenty-two species are currently known from the Americas, and of those, seven have been reported from Eastern Tropical America: *Perinereis anderssoni* Kinberg, 1865 described from Brazil; *Perinereis cariacoensis* Liñero-Arana, 1983 and *Perinereis mochimaensis* Liñero-Arana, 1983 from Venezuela; *Perinereis cariboea* de León-González & olís-Weiss, 1998 from Mexican Caribbean, *Perinereis floridana* Ehlers, 1868 from Florida, USA, *Perinereis ponteni* Kinberg, 1865 from Brazil, and *Perinereis vancaurica* Ehlers, 1868 from Thailand. The latter is reported for the region by many authors, including Fauvel (1919) from French Guiana, Wesenberg-Lund (1958) from Bonaire, Lana (1984) from Pontal do Sul and Pecas Island, Brazil, and Santos and Lana (2000) from Brazil.

In the present work, a new species of *Perinereis* from the west coast of Florida is described for the first time. Further, records of Indo-Pacific nereidids reported from the Americas, such as *Perinereis vancaurica*, are evaluated.

## Material and methods

The material analyzed for this study was collected in December 2010 by Florida Atlantic University, Department of Biological Sciences/Harbor Branch Oceanographic Institute from natural oyster reefs within Rookery Bay National Estuarine Research Reserve near Naples, Florida. Sampling in Rookery Bay took place as part of the BP Oil Spill Oyster Study, an impact study on the effects of an oil spill on the oyster reef systems in Florida, funded by the Florida Institute of Oceanography. Samples were hand collected at low tide using a 0.12 m² collection quad to a depth of 7.6 cm. The materials were screened through a 2.0 mm mesh sieve. The collection team, led by Dr Donna Devlin, included Dr Holly Nance, Dr Loren Coen, Pedro Lara, and Dana Smith. Specimens were fixed with formalin and preserved in 70% isopropyl alcohol, no stain was used. Material was deposited in the Los Angeles County Museum, Allan Hancock Foundation (LACM-AHF), the Zoologische Museum, Hamburg, Germany (HZM), the Polychaetological Collection of the Universidad Autónoma de Nuevo León (UANL), and the Florida Fish and Wildlife Conservation Commission – Fish and Wildlife Institute Invertebrate Collection, St. Petersburg, Florida (FSBC I). A key to all known American species of *Perinereis* is included.

## Results

### Systematics Class Polychaeta Grube, 1850 Order Phyllodocida Örsted, 1843 Family Nereididae Blainville, 1818 Genus *Perinereis* Kinberg, 1865

#### Perinereis rookeri sp. n.

urn:lsid:zoobank.org:act:6177521A-A7AF-43E7-9C82-57A39E408436

http://species-id.net/wiki/Perinereis_rookeri

Figures 1 , 2

##### Type material.

West coast of Florida, Naples, Rookery Bay, December 17-22, 2010 (holotype, UANL 7841), and 3 paratypes (HZM P-27422), FLW-Site3-Reef5-Rep2, 26°00.56'N, 81°44.90'W, 22/12/2010; One Paratype (LACM-AHF-4998), FLW-Site1-Reef2-Rep1, 26°01.55'N, 81°44.00'W, 17/12/2010; One Paratype (UANL 7842), FLW-Site3-Reef3-Rep2, 26°00.58'N, 81°44.24'W, 21/12/2010.

**Additional material.**

West Coast of Florida, Naples, Rookery Bay, December 17–21, 2010. One specimen (FSBC I 106475) from FLW-Site1-Reef3-Rep2, 26°01.54'N, 81°44.23'W, 17/12/2010; and one specimen from FLW-Site3-Reef3-Rep2, 26°00.58'N, 81°44.24'W, 21/12/2010.

##### Description.

Holotype complete, 84 chaetigers, 52 mm in length, 2.5 mm wide at chaetiger 10 (excluding parapodia); 3.72 mm wide at chaetiger 10 (including parapodia). Paratypes complete with 75-87 chaetigers, 42-65 mm long, and 0.8–2.7 mm wide at chaetiger 10 (excluding parapodia).

**Figure 1.**
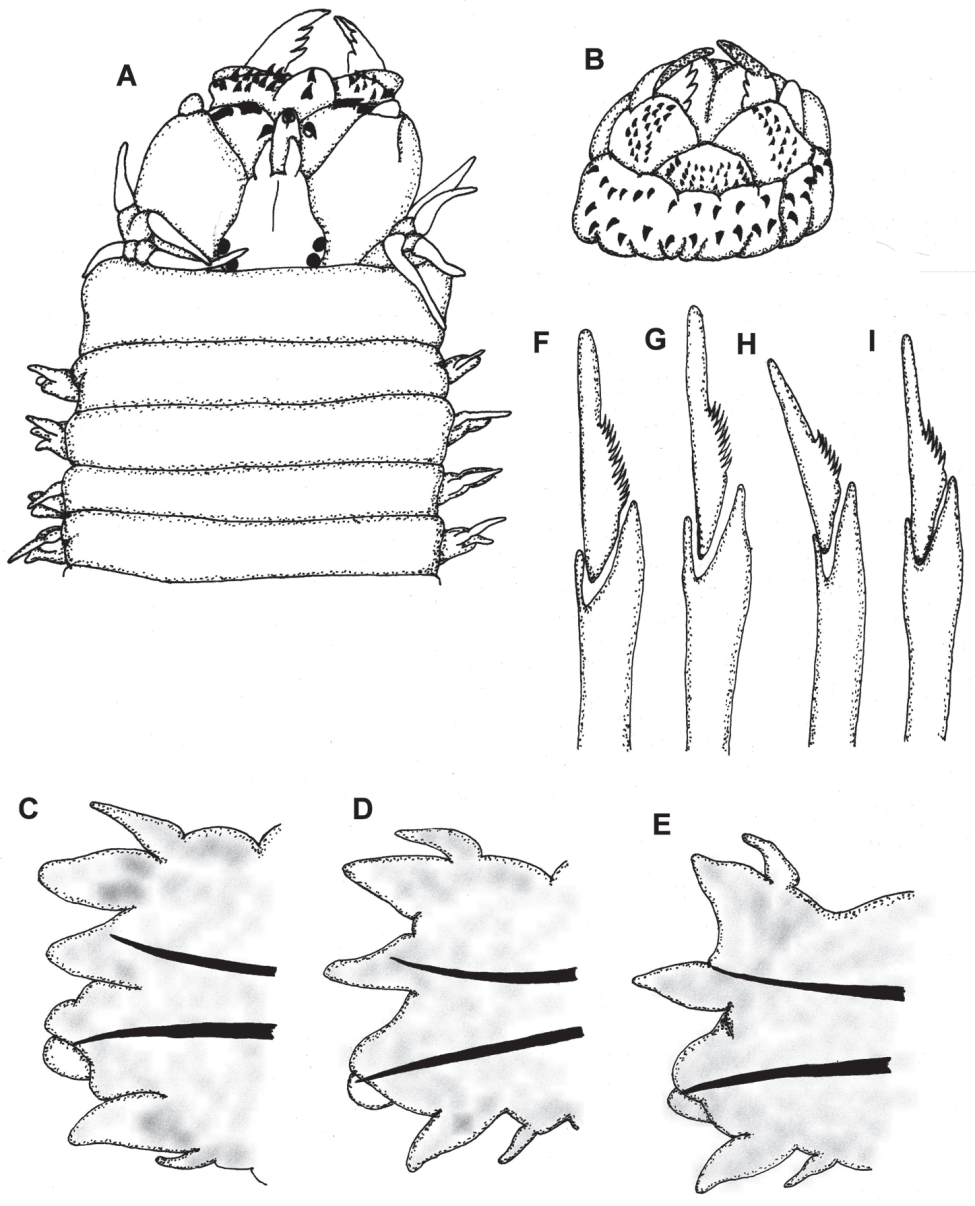
*Perinereis rookeri* sp. n. **A** Anterior end, dorsal view **B** Proboscis, ventral view **C–E** Parapodia of chaetigers 11, 30 and 61, anterior view **F** Supracicular neuropodial heterogomph falciger, chaetiger 11 **G** Infracicular neuropodial heterogomph falciger, chaetiger 11 **H** Supracicular neuropodial heterogomph falciger, chaetiger 61 **I** Infracicular neuropodial heterogomph falciger, chaetiger 61. Measures: **A–B** = 1 mm; **C–E** = 250µ; **F–I** = 30µ.

Prostomium slightly wider than long, antennae minute, about ⅓ length of prostomium. Two pairs of eyes of similar size in trapezoidal arrangement. Biarticulate palps globose, with four pairs of tentacular cirri, posterodorsal pair extending back to posterior margin of first chaetiger (Fig. 1A).

**Figure 2.**
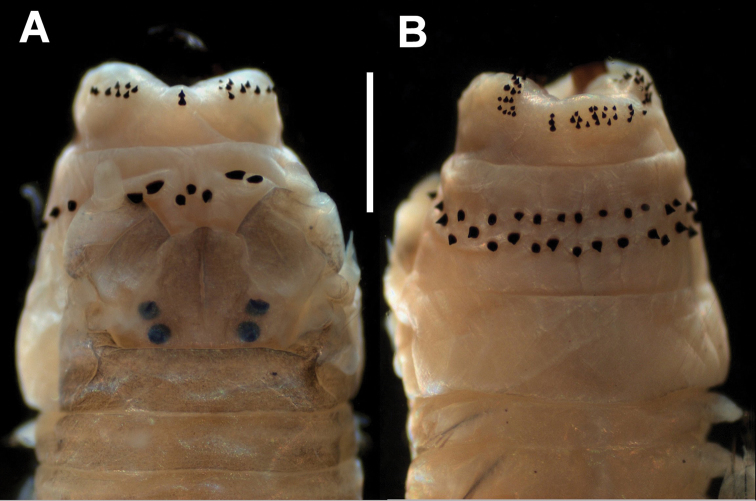
*Perinereis rookeri* sp. n. **A** Anterior end, dorsal view **B** Anterior end, ventral view. Measures: **A–B** = 1 mm.

Paragnaths black, cones on maxillary ring, cones and bars on oral ring, those of maxillary ring smaller. Area I = 2 cones in a line; Area II = 13 cones in 3 irregular rows; Area III = 18 cones in a quadrangular arrangement of 3 irregular rows, flanked by a left line of 3 cones and a right line of 5 cones; Area IV = 20 cones on the left, 17 cones on the right in a triangular patch, without bars; Area V = 3 cones in a triangle; Area VI = two short transverse bars; Area VII-VIII = 33 cones in two rows, basal row with slightly longer paragnaths in relation to those of the distal row (Fig. 1A–B, 2A–B).

First two parapodia uniramous, all others biramous. Parapodia of anterior region with short dorsal cirri, not longer than dorsal ligule, inserted basally, dorsal ligule subulate, notopodial ventral ligule subtriangular, without notopodial prechaetal lobe; neuropodia with superior lobe rounded, inferior lobe reduced, postchaetal lobe rounded, ventral ligule subulate, ventral cirri minute, inserted basally (Fig. 1C). Parapodia of median and posterior region similar in shape, notopodia with short dorsal cirri inserted medially, dorsal ligule subtriangular, notopodial ventral ligule subulate (Fig. 1D–E); neuropodial structures similar in shape along body.

Chaetation similar throughout body. Notochaetae all homogomph spinigers. Supracicular neurochaetae consisting of homogomph spinigers and heterogomph falcigers, the latter with straight blades denticulate on the basal half (Fig. 1F–G). Falciger blades of anterior and median parapodia longer than those of posterior ones. Infracicular neurochaetae consisting of heterogomph spinigers and heterogomph falcigers, the latter similar in shape and size gradation of dentition to supracicular ones (Fig. 1H–I). Anterior and median spinigers slightly longer than posterior ones.

Pygidium with terminal anus and a pair of short cirri (1.2 mm long) inserted ventrally to anal opening.

##### Remarks.

Type and non-type specimens exhibit variation in paragnath counts for Area I, as follows. A single specimen with 1 paragnath, two specimens with 3 in a triangle, and the rest with 2 paragnaths in a line.

##### Discussion.

Hutchings et al. (1991) created an informal grouping of species belong to *Perinereis* based on the ornamentation of pharyngeal Area VI, and the development of the dorsal ligule. The species described here belongs to group 2A based on the presence of two short bars on Area VI and a dorsal ligule that is not greatly expanded. The following species belong to this group: *Perinereis aibuhitensis* (Grube, 1878) from the Philippines, *Perinereis brevicirrata* (Treadwell, 1920) from Southern Brazil, *Perinereis camiguinoides* (Augener, 1922) from Juan Fernandez Island, *Perinereis jascooki* Gibbs, 1972 from the Cook Islands, *Perinereis kuwaitensis* Mohammad, 1970 from Kuwait, *Perinereis singaporiensis* (Grube, 1878) from Singapore, and *Perinereis vancaurica* (Ehlers, 1868) from Nicobar Islands.

This group can be further subdivided by the presence or size of the notopodial prechaetal lobe. *Perinereis aibuhitensis*, *Perinereis jascooki*, *Perinereis kuwaitensis*, and *Perinereis singaporiensis*, possess only dorsal and median ligules, and the notopodial prechaetal lobe is reduced or absent. The absence of a notopodial prechaetal lobe places *Perinereis rookeri* sp. n. within this subgroup. *Perinereis rookeri* sp. n. and *Perinereis singaporiensis* are the most similar; both species have Area III with a rectangular group of small paragnaths arranged in 4 irregular rows, flanked by 8 cones in two vertical lines. The two species differ by the length of the tentacular cirri, the shape of the dorsal ligule, and the dentition on the inner margin of the falciger blades. The longest tentacular cirrus of *Perinereis singaporiensis* reaches chaetiger 4, while on *Perinereis rookeri* sp. n. it reaches chaetiger 1. The dorsal notopodial ligule on *Perinereis singaporiensis* is conical anteriorly and triangular on median and posterior notopodia, *Perinereis rookeri* sp. n. has subulate dorsal ligules on anterior notopodia and subtriangular on median and posterior notopodia. The falciger blades on *Perinereis singaporiensis* are subtriangular and denticulate on ¾ of their length, whereas those of *Perinereis rookeri* sp. n. are subulate and denticulate for only ½ of their length. The data listed here were taken based on Hutchings et al. (1991), who reviewed the holotype of *Perinereis singaporiensis*. They note that this material is in very poor condition, however the pharyngeal arrangement, shape of the falcigers and number of chaetal lobes agree with their description. The original description from Grube (1878) differs from the Australian specimens in that the longest tentacular cirrus extends to segment 5 and in the pharyngeal arrangement: Area I = 2 cones in line; Area III = 23 cones in transverse group with 2 cones on each side; Area V = 1 cone.

The range of *Perinereis brevicirrata* is the most geographically proximate to that of *Perinereis rookeri* sp. n., but the two can be readily distinguished morphologically. *Perinereis brevicirrata* has notopodial prechaetal lobes, Area V has 2 large cones, and Area VII-VIII has 3 lines of paragnaths. In *Perinereis rookeri* sp. n.notopodial prechaetal lobes are absent, Area V has 3 cones in a triangle, and Area VII-VIII has two well-defined lines of 33 paragnaths.

It is likely that *Perinereis rookeri* sp. n. specimens have been previously misidentified or grouped into a higher taxonomic group during previous benthic macroinvertebrate studies along the west coast of Florida.

Many studies throughout southwestern Florida collected only one species of *Perinereis*, *Perinereis floridana*, including a 1932 survey conducted around the Dry Tortugas (Monro 1933) and a 1963 to 1969 Tampa Bay study, which included oyster reef sampling sites (Taylor 1971). Many subsequent studies from southwestern Florida showed an absence of *Perinereis* species completely, including a 1957-1960 estuarine ecology study in north Florida Bay (Tabb and Manning 1961). No *Perinereis* species were reported from within Rookery Bay during a 1984 to 1985 study; however, specimens labeled as *Nereis* or *Neanthes* sp. A were documented. The research also noted a lack of previous benthic macroinvertebrate research conducted specifically within Rookery Bay (Thoemke and Gyorkos 1988). A 2006 study focused on epifaunal community development associated with artificial oyster reefs created near St. Petersburg also found no *Perinereis* specimens, although four unidentified Annelida species were reported (Dow 2008). In northwestern Florida, during a systematic faunal inventory within Pensacola Bay from 1961-1963, three species of *Perinereis* were recorded, including *Perinereis andersonni*, *Perinereis floridana*, and an unidentified *Perinereis* species. All three species were considered rare, found only during the winter of 1962-1963 in areas with salinity at 20 ppt or greater and within a sandy-mud benthic habitat (Cooley 1978). Taxonomic checklists for Florida list *Perinereis andersonni* and *Perinereis floridana* as the only two species of *Perinereis* found within Florida waters (Perkins and Savage 1975, Camp et al. 1998, Fauchald et al. 2009).

Due to the probable association *Perinereis rookeri* sp. n. has with oyster reefs, it is likely that any occurrence in previous studies would have been rare. *Perinereis rookeri* sp. n. specimens, if previously collected, have presumably been misidentified or left at a higher taxonomic level in earlier research from Florida’s west coast.

##### Etymology.

This specific name is derived from Rookery Bay National Estuarine Research Reserve on the west coast of Florida where the species was first discovered.

##### Distribution.

This species is known from the Gulf of Mexico on the west coast of Florida, within Rookery Bay National Estuarine Research Reserve near Naples, where it was collected in association with oyster reefs in estuarine environments.

##### Ecological comments.

*Perinereis rookeri* sp. n. was collected solely from oyster reefs within Rookery Bay National Estuarine Research Reserve. The oyster reefs located in Rookery Bay are shallow, intertidal reefs located in water depths of 1.2-1.5 m at high tide, with approximately 80% of the reefs exposed during low tide. Salinity at the sites where this species was found ranged from 35 to 37 ppt.

### Key to American species of *Perinereis*

**Table:** 

1	Area VI with more than 2 bars	2
–	Area VI with no more than 2 bars	4
2	Posterior parapodia with dorsal ligule expanded; maxillary ring with few paragnaths (Area I and III without paragnaths); Area V with 1 paragnath; Area VI with 16 small paragnaths in a line	*Perinereis seridentata* (Hartmann-Schröder, 1959)
–	Posterior parapodia without dorsal ligule expanded; numerous paragnaths in the maxillary ring	3
3	Area IV with bars; Area V with 1–2 paragnaths; Area VI with 12 to 16 small bars in a transverse line	*Perinereis vallata* (Grube, 1857)
–	Area IV without bars; Area V with 3 paragnaths; Area VI with 5 to 10 small cones in a transverse line	*Perinereis gualpensis* Jeldes, 1961
4	Posterior parapodia with dorsal ligule not expanded	5
–	Posterior parapodia with dorsal ligule expanded	8
5	Notopodial prechaetal lobe present on anterior and median notopodia	7
–	Notopodial prechaetal lobe absent	6
6	Area VI with 1 transverse bar; Area V with 1-2 paragnaths in line; longest tentacular cirri reaching up to chaetiger 11	*Perinereis floridana* Ehlers, 1868
–	Area VI with 2 bars; Area V with 3 paragnaths in triangle; longest tentacular cirri reaching chaetiger 1	*Perinereis rookeri* sp. n.
7	Neuropodial heterogomph falcigers with short blades, with teeth on 3/4 of the inner edge. Longest tentacular cirri reaching chaetiger 3 to 5; Area I with 1 paragnath; Area V with 3 paragnaths	*Perinereis camiguinoides* (Augener, 1922)
–	Neuropodial heterogomph falcigers with long blades, with teeth on 1/2 of the inner edge; longest tentacular cirri not exceed the length of the prostomium; Areas I and V with 2 paragnaths in a line	*Perinereis brevicirrata* (Treadwell, 1920)
8	Area VI with 2 transverse bars	9
–	Area VI with 1 transverse bar or a big conical paragnath	11
9	Area VII-VIII with 8 paragnaths in a line; Area I with 4 paragnaths in a diamond; Area V with 1 paragnath	*Perinereis osoriotafalli* de León-González & Solís-Weiss, 1998
–	Area VII-VII with two lines of paragnaths	10
10	Area I with 2 paragnaths in a line; Area III with 7 paragnaths in an oval group; Area V bare; neuropodial heterogomph falcigers with short blades	*Perinereis cariboea* de León-González & Solís-Weiss, 1998
–	Area I with 11 paragnaths in a group; Area III with 17 paragnaths in an oval group; Area V with 2 paragnaths; neuropodial heterogomph falcigers with long blades	*Perinereis mochimaensis* Liñero-Arana, 1983^1^
11	Area VI with a short transverse bar or a big paragnath	12
–	Area VI with a long transverse bar	16
12	Area VI with a big cone shaped paragnath	13
–	Area VI with a short transverse bar	14
13	Anterior notopodia with notopodial prechaetal lobe; Area I with 1 paragnath; Area IV with 2 bars	*Perinereis monterea* (Chamberlin, 1918)
–	Notopodia without notopodial prechaetal lobe; Area I with 2 paragnaths in line and numerous small paragnaths on each side; Area IV without bars	*Perinereis falklandica* (Ramsay, 1914)
14	Area IV usually with bars; notopodial prechaetal lobe absent; Area V with 3 paragnaths in triangle; Area VI with a short, straight bar	*Perinereis pseudocamiguina* (Augener, 1922)
–	Area IV without bars, only paragnaths; notopodial prechaetal lobe may be present	15
15	Notopodial prechaetal lobe evident on posterior notopodia; tentacular cirri long, reaching chaetiger 6–7; Area I and V with 1 paragnath	*Perinereis villalobosi* Rioja, 1947
–	Notopodial prechaetal lobe absent; tentacular cirri short, reaching chaetiger 1; Area I with 4 paragnaths in a diamond; Area V with 3 paragnaths in a triangle	*Perinereis anderssoni* Kinberg, 1865
16	Area I with 2–3 paragnaths in line; Area V with 3 paragnaths in a triangle	17
–	Area I with a large group of paragnaths; Area V with 1 paragnath	18
17	Tentacular cirri short, reaching chaetiger 2; neuropodial heterogomph falcigers with short blades	*Perinereis longidonta* Rozbaczylo & Castilla, 1973
–	Tentacular cirri long, reaching chaetiger 7 to 9; neuropodial heterogomph falcigers with long blades	*Perinereis helleri* (Grube, 1878)^2^
18	Area VII–VIII with 7 small paragnaths in one line; Area I with a group of 7 paragnaths	*Perinereis bajacalifornica* de León-González & Solís-Weiss, 1998
–	Area VII–VIII with paragnaths in two lines	19
19	Area I with an oval group of 11 paragnaths; dorsal cirri inserted medially on posterior parapodia; falcigers with a distal tooth directed down	*Perinereis elenacosoae* Rioja, 1947
–	Area I with a triangular group of 8 paragnaths; dorsal cirri inserted subdistally on posterior parapodia; falcigers distally pointed	*Perinereis ponteni* Kinberg, 1865^3^

**Notes**

1 *Perinereis mochimaensis* Liñero-Arana, 1983 and *Perinereis cariacoensis* Liñero-Arana, 1983 differ in that *Perinereis mochimaensis* has 5 transverse paragnaths in areas V and VI and posterior notopodial superior lobe larger. However, only one specimen of each was examined, one of them incomplete, and these are the only differences indicated. Therefore, until a revision is carried out we consider that *Perinereis cariacoensis* could be a junior synonym of *Perinereis mochimaensis*, which is why it was not included in the key.

2 *Perinereis helleri* was described by Grube (1878) from the Philippines and was reported from Pascua Island, Chile by Rozbaczylo and Castilla (1973). The characters described for the Chilean specimens, such as pharyngeal arrangement, are similar to those from the Philippines and Australia, however, the descriptions of Pascua Island´s specimens were incomplete and were not accompanied by any illustration. Consequently, the presence of *Perinereis helleri* in the Americas remains doubtful.

3 *Perinereis ponteni* Kinberg, 1865, was described from Rio de Janeiro, Brazil, the description was brief and very general. Hartman (1948) proposes a doubtful synonymy with *Perinereis anderssoni*, however, both species can be easily distinguished by the presence of a long, ribbon shaped bar on the Area VI of *Perinereis ponteni*, and a short bar on the same area in *Perinereis anderssoni*. After the revision of one of the two existent syntypes, we consider that *Perinereis ponteni* is valid.

**Additional notes**

*Perinereis obfuscata* was described from the Philippines by Grube (1878). Its occurrence has been documented from many coasts throughout the world, with great variability of morphological characters. The record of Rioja (1941) for western Mexico does not belong to this species; rather this record as well as that of Berkeley and Berkeley (1960) both from Guerrero, Mexico belong to *Perinereis elenacasoae* Rioja, 1947 (de León-González and Solís-Weiss 1998). For that reason *Perinereis obfuscata* was not included in the key.

*Perinereis vancaurica* was originally described from the Nicobar Islands, Andaman Sea. The records for America are limited to the Atlantic (Fauvel 1919, Wesenberg-Lund 1958, Lana 1984, Santos and Lana 2000); however, the descriptions and illustrations provided in those works are insufficient to verify its presence in American waters. Lana (1984) provides a description and a figure of a median parapodium, but with only this information it is impossible to verify whether Lana´s specimens belongs to the Andaman Sea species; furthermore, in the discussion section Lana mentioned that *Perinereis brevicirrata* could be similar to *Perinereis vancaurica*, but both species can be distinguished clearly by the presence of notopodial prechaetal lobe in *Perinereis brevicirrata* and the absence of the same lobe in *Perinereis vancaurica*. For that reason *Perinereis vancaurica* was not included in the key.

## Supplementary Material

XML Treatment for Perinereis rookeri
